# Guy's Hospital and the Calling-Up of Doctors

**Published:** 1917-07-07

**Authors:** 


### GUY'S HOSPITAL AND THE CALLING-UP OF DOCTORS.

### Correspondence with the Local Government Board.

A COMMUNICATION has been addressed to the President of the Local Government Board by Mr. H. Cosmo Bonsor, the President, Mr. Whitbred, and Sir Cooper Perry, on behalf of the governors of Guy's Hospital, drawing attention to the effect on the hospitals of the calling-up of doctors, and the difficulties in which hospitals with medical schools are likely to be placed unless steps are taken to prevent the further depletion of their staffs. Stress is further laid upon the fact that, owing to the already existing diminution in the *personnel* of the staffs and the small number of students available for rendering assistance, some specialists of military age are now almost single-handed, carrying on the entire work of their important departments. It is pointed out that, if the specialists in question be absorbed into the Army, there will be no alternative but to shut down entirely some of these departments, to the great detriment of the patients now attending them; and it is asked that the Government shall give a clear indication that it is their patriotic duty to continue their present service to the civil population.

LORD RHONDDA'S ANSWER.

In the course of his reply, dated Local Government Board, 18th ultimo, Lord Rhondda writes:—

"I gladly acknowledge the great value of the services which the hospitals render to the Community, and I am most anxious that nothing should be done to hinder the work which they are now carrying on in the public interest.

"I am, of course, aware of the difficult position in which the hospitals are placed at the present time, but I hope that the provision which has already been made in pursuance of the Military Service Act, 1916, will prevent any undue hardship being caused to these institutions. As you are aware, a Committee of Reference, appointed by the Royal College of Physicians and the Royal College of Surgeons, has been set up to deal with members of the staffs of hospitals, and I hope that they will make arrangements for meeting the needs of the Army without unduly restricting hospital services.

"I understand that Lord Derby, whilst reserving to himself the right to reconsider the position in the event of the requisite number of doctors not being obtained for the Army by the present machinery, has agreed that for the present he will not grant commissions to doctors except on the recommendation of the Central Medical War Committee. This arrangement will prevent any member of a hospital staff being taken until his case has been considered by the Committee of Reference.

"Perhaps I may add that while I fully realise the patriotism exhibited by the medical and surgical staffs of the hospitals in their readiness to undertake military duties, I earnestly hope that specialists and others who are responsible for essential departments of the large hospitals—*e.g.*, the treatment of Children's Diseases—will carefully consider the claims of their hospitals and the importance of the work they are there doing for the country. The maintenance of the health of the population at home is a matter of vital necessity."

